# Behavior Change Content, Understandability, and Actionability of Chronic Condition Self-Management Apps Available in France: Systematic Search and Evaluation

**DOI:** 10.2196/13494

**Published:** 2019-08-26

**Authors:** Luiza Siqueira do Prado, Camille Carpentier, Marie Preau, Anne-Marie Schott, Alexandra Lelia Dima

**Affiliations:** 1 Équipe d'Accueil 7425 Health Services and Performance Research Université de Lyon Lyon France; 2 Équipe d'Accueil 4163 Groupe de Recherche en Psychologie Sociale Psychology Institute Université Lumière Lyon 2 Bron France; 3 Centre Hospitalier Lyon Sud Pôle de Santé Publique Hospices Civils de Lyon Lyon France

**Keywords:** mHealth, mobile phone, app, self-management, chronic conditions, target behaviors, behavior change techniques, understandability, actionability

## Abstract

**Background:**

The quality of life of people living with chronic conditions is highly dependent on self-management behaviors. Mobile health (mHealth) apps could facilitate self-management and thus help improve population health. To achieve their potential, apps need to target specific behaviors with appropriate techniques that support change and do so in a way that allows users to understand and act upon the content with which they interact.

**Objective:**

Our objective was to identify apps targeted toward the self-management of chronic conditions and that are available in France. We aimed to examine what target behaviors and behavior change techniques (BCTs) they include, their level of understandability and actionability, and the associations between these characteristics.

**Methods:**

We extracted data from the Google Play store on apps labelled as *Top* in the *Medicine* category. We also extracted data on apps that were found through 12 popular terms (ie, keywords) for the four most common chronic condition groups—cardiovascular diseases, cancers, respiratory diseases, and diabetes—along with apps identified through a literature search. We selected and downloaded native Android apps available in French for the self-management of any chronic condition in one of the four groups and extracted background characteristics (eg, stars and number of ratings), coded the presence of target behaviors and BCTs using the BCT taxonomy, and coded the understandability and actionability of apps using the Patient Education Material Assessment Tool for audiovisual materials (PEMAT-A/V). We performed descriptive statistics and bivariate statistical tests.

**Results:**

A total of 44 distinct native apps were available for download in France and in French: 39 (89%) were found via the Google Play store and 5 (11%) were found via literature search. A total of 19 (43%) apps were for diabetes, 10 for cardiovascular diseases (23%), 8 for more than one condition in the four groups (18%), 6 for respiratory diseases (14%), and 1 for cancer (2%). The median number of target behaviors per app was 2 (range 0-7) and of BCTs per app was 3 (range 0-12). The most common BCT was *self-monitoring of outcome(s) of behavior* (31 apps), while the most common target behavior was *tracking symptoms* (30 apps). The median level of understandability was 42% and of actionability was 0%. Apps with more target behaviors and more BCTs were also more understandable (ρ=.31, *P*=.04 and ρ=.35, *P*=.02, respectively), but were not significantly more actionable (ρ=.24, *P*=.12 and ρ=.29, *P*=.054, respectively).

**Conclusions:**

These apps target few behaviors and include few BCTs, limiting their potential for behavior change. While content is moderately understandable, clear instructions on when and how to act are uncommon. Developers need to work closely with health professionals, users, and behavior change experts to improve content and format so apps can better support patients in coping with chronic conditions. Developers may use these criteria for assessing content and format to guide app development and evaluation of app performance.

**Trial Registration:**

PROSPERO CRD42018094012; https://www.crd.york.ac.uk/prospero/display_record.php?RecordID=94012

## Introduction

Chronic conditions are the main cause of disability and premature death worldwide, representing the highest number of disability-adjusted life years in the Global Burden of Disease project [1]. More specifically, four groups of diseases—cardiovascular diseases, cancers, respiratory diseases, and diabetes—cause 80% of premature deaths related to chronic conditions [2]. The rise in prevalence of such conditions, while being determined by multiple causes, is highly related to unhealthy lifestyles and population aging. Treatment requires a long-term and multidisciplinary approach, including therapeutic education and lifestyle changes, to prevent further aggravation and/or premature death; treatment focuses on modifiable behavioral risk factors, such as insufficient physical activity or inadequate diet [2]. Achieving and maintaining satisfactory quality of life is strongly dependent on the patient’s ability to reduce behavioral risks and to regularly perform specific self-care activities defined together with health care providers (ie, self-management behaviors). This process of active engagement in obtaining skills and taking part in health-related decisions is also called patient empowerment [3]; this may be mediated by technology that facilitates self-awareness and understanding how and when to take action regarding measured physiological parameters, to prevent or react to deterioration in health status.

Currently, mobile health (mHealth) mobile phone apps can support patients in performing behaviors such as symptom monitoring and medication intake, among others [4]; therefore, they have the potential to help improve population health and reduce health care costs. By the end of 2017, mobile broadband subscriptions were expected to reach 4.3 billion worldwide [5]. In 2012, one in five mobile phone users had at least one health-related mobile app on his or her phone [6]; in 2015, over half of that population had downloaded at least once a health-related app [7]. In addition, numbers of mHealth apps downloaded are similar between individuals with and without chronic conditions [8]. The availability of mHealth apps and people’s tendency to carry their devices with them at all times mean they can also be used for delivering behavioral interventions to large populations [9]. Yet, despite the increasing number of studies and reviews on the use of such apps on health outcomes, evidence for effectiveness is still unclear [10,11]. Furthermore, in the fast-paced technology culture, few mHealth interventions are designed in collaboration with patients, clinicians, or behavioral scientists or are subject to rigorous testing [4,12]. The result is a wide and heterogeneous range of offerings that differ in their objective, content, and user experience [13].

To assess the potential of self-management apps to improve individual and population health, it is useful to consider them as technology-mediated health behavior change interventions. For such interventions to be effective, they need to intervene on the causal behavioral pathways relevant to the health of their user group (ie, to target specific behaviors causally linked to the desired outcomes). Moreover, from a psychological perspective, they need to include behavior change techniques (BCTs), which are the *active ingredients* of behavior change interventions: reproducible and irreducible components of these interventions that can trigger change in the psychological determinants of these behaviors and, consequently, improve health [14]. In recent years, health behavior theorists and intervention developers have been building consensus on methods to identify BCTs present in existing interventions, which resulted in a 93-BCT taxonomy that is currently used as a shared framework for intervention evaluation and development [15]. The presence of behavioral change content has been shown to increase the effectiveness of mHealth and Internet-based interventions [16,17]. Examining the mHealth app offerings in terms of occurrence of target behaviors and BCTs can be informative regarding the current state-of-the-art on behavior change; it can also highlight opportunities for improvement [18], for example, by studying links between usage patterns of individual behavior change content and changes in health outcomes.

The presence of relevant behavior change content does not by itself guarantee that users will be able to interact with it and potentially change their behaviors. These tools need to present information in an accurate, comprehensible, and actionable way; they must also consider different communication competencies, styles, and health literacy levels to optimize their reach and enhance health decision making [19]. Evidence shows that most educational materials are too complex for patients with low health literacy [20]. To assess the suitability of mHealth apps for diverse audiences, it is useful to consider them as health-related materials. The Patient Education Material Assessment Tool for audiovisual materials (PEMAT-A/V) is a commonly used method in this domain; this tool evaluates the extent to which health-related materials are understandable (ie, understandability) and give clear instructions regarding actions that users may take to apply the information presented (ie, actionability) [20]. Presenting relevant behavior change content in a suitable manner is therefore important for ensuring that the intended users achieve the goals the app is supposed to facilitate. Apps with richer behavioral content may also present it in a more understandable and actionable way, and this can be seen as an indicator of app quality and of the level of expertise of the developing team. Yet, to our knowledge, no examination of both content and suitability of apps was performed to date. Understanding the links between content and format in the current app offerings in a specific territory may provide insights into the rapidly evolving app development phenomenon and recommendations for improvement.

As mHealth develops worldwide, evaluations of app content and format are increasingly common and necessary to inform policy discussions on achieving the potential of mHealth to improve public health [21]. For example, BCTs were shown not to be widely implemented in top-ranked physical activity apps in the United States [22]. In New Zealand, in physical activity and dietary apps, BCTs associated with increased effectiveness in modifying these behaviors were more common in paid apps [23]. In Canada, theory-based cognitive-behavioral content was found to be present in only 10% of apps for depression [24]. Although app use is a global phenomenon, research is normally limited to apps in English, while the available offerings in commercial marketplaces are restricted to geographical regions. The French Health National Strategy 2018-2022 has forecasted a generalization of digital services in health care and put special interest in promoting favorable health behaviors and fighting social inequities in access to health care [25]. It is thus important to assess the current mHealth app offerings in France and in French, especially for self-management of chronic conditions. To our knowledge, no review with these characteristics has been published to date worldwide, and the potential of these tools to support behavior change for self-management of chronic conditions has not yet been fully examined. Such evaluation is instrumental for orienting the development of this expanding field in a way that best serves the interests of all stakeholders, including patients, health care professionals, app developers, payers, and the health care system.

Therefore, this systematic review of mHealth apps for chronic condition self-management in France aimed to answer the following questions: (1) What behaviors are targeted in these apps, and by which BCTs?; (2) What levels of understandability and actionability characterize these apps?; and (3) Are apps with more behavioral change content also easier to understand and act upon?

## Methods

### Overview

We developed a systematic review protocol based on the Preferred Reporting Items for Systematic Reviews and Meta-Analyses Protocols (PRISMA-P) guidelines, an evidence-based minimum set of items for reporting systematic reviews [26]. We registered the protocol with the International Prospective Register of Systematic Reviews (PROSPERO), an international database of prospectively registered systematic reviews (registration number: CRD42018094012). The PRISMA checklist [26] is available in Multimedia Appendix 1.

Apps were identified through two different approaches: (1) a search of peer-reviewed articles reporting on development or validation of mHealth apps for self-management of chronic conditions and (2) a search in the Android commercial marketplace for mobile phones. We used the Android marketplace Google Play store as it represents 88% of the global mobile phone market [27] and most apps are developed for both operating systems (ie, Android and iOS).

A systematic search of PubMed (ie, MEDLINE), IEEE, and Web of Science electronic bibliographic databases was conducted; all search terms are available in Multimedia Appendix 2. We searched for peer-reviewed articles and conference papers published between 2012 and 2018 concerning mHealth self-management interventions for the previously stated chronic conditions. Articles and papers had to report on empirical research on the development or validation of mHealth tools, pilot studies, or randomized controlled trials, both protocols and reports of study results. Articles and papers were assessed independently by two investigators (LSdP and CC) based on title and abstract, followed by full-text examination to identify available apps in France, in French, and for Android from the Google Play store.

Subsequently, a list of the first 500 free apps labelled as *Top* in the Google Play store in the *Medicine* category and the first 55 paid apps available in the same category was extracted (n=555). The number of paid apps was limited by the marketplace. Another search was performed using 12 keywords in French related to the four groups of diseases: cardiovascular diseases, cancers, respiratory diseases, and diabetes. The keywords were as follows: “maladie cardiaque,” “maladie coeur,” “AVC accident vascular cérébral,” “infarctus,” “maladie pulmon,” “asthme,” “BPCO,” “maladie respiratoire,” “cancer,” “diabète,” “diabète type 1,” and “diabète type 2.” For each keyword, the first 20 apps shown in the marketplace (n=240) were extracted. The complete list (n=795) was assessed independently by two investigators. After screening, selected apps were divided into five groups: four according to the conditions they targeted— *cancers, diabetes, respiratory diseases*, or *cardiovascular diseases—* and one generic group (ie, *other*) for apps that targeted behaviors like medication adherence and physical activity, irrespective of medical condition (ie, they did not specifically reference a disease within the previous four groups).

All searches, data extraction, and coding were done between March and April 2018. Coding was performed a second time in October 2018 by a different investigator, along with interrater reliability, reconciliation, and coding review. A Samsung J7 mobile phone with Android, version 6.0.1, was used for downloading and evaluating all selected apps.

### Eligibility

All native apps available for download in France and in French at the Google Play store designed for patients for the self-management of cardiovascular diseases, cancers, respiratory diseases, and diabetes were eligible. There was no restriction considering price or the source of for-profit or not-for-profit funding. The following apps were excluded: apps that were clearly not for chronic conditions (ie, apps that did not state their main purpose or were designed for other users or purposes, such as training health professionals or students, hospitals, or medical laboratories; making medical appointments; reaching emergency services; or geographical localization of defibrillators or pharmacies); apps for chronic conditions other than those in the four groups studied in this work; apps for chronic conditions in the four groups that were not for self-management (ie, offering general health information or risk assessment); apps that required additional hardware, like special glucometers; apps with descriptions in French but content in English or with different names and same content; and, finally, apps that were no longer available in October 2018.

Eligible apps may describe their objective as chronic disease self-management or target only specific behaviors relevant for these conditions, such as medication adherence, trigger management, exacerbation management, physical activity, dietary behaviors, and archiving health information. Apps that targeted only preventive behaviors, such as physical activity and diet, with no reference to chronic condition management were also excluded.

### Screening and Selection

The reference management software, Zotero (Corporation for Digital Scholarship), was used to identify and remove duplicate records in the literature search. Titles and abstracts of remaining records were screened by two independent reviewers to establish eligibility. If two reviewers recommended study or app inclusion, the full text of the study or the app availability was sourced for review and appraisal in order to determine eligibility for this study. If reviewer discordance arose, consensus was reached through discussion and arbitration with a third investigator.

### Data Extraction and Analysis

The following app characteristics were extracted from the Google Play store, along with app’s name and available description: information on the presence of sales on the app (ie, paid app or free app with or without paid features), number of downloads (ie, from 10+ to 10,000,000+), user ranking (ie, from 1 to 5 stars), number of ratings, version, last update, and developer information. Developers were then categorized into three groups: (1) *private company*, comprising single-app developers and dedicated app-developing companies; (2) *nonprivate*, comprising nongovernmental organizations, public institutions, or European projects; and (3) *pharmaceutical and medical device companies*, comprising bigger players in the market, such as pharmaceutical laboratories and other medical technology companies.

### Target Behaviors and Behavior Change Content

Target behaviors were coded following detailed examination of app content and further functions, like sending notifications to users to perform tasks such as drinking water, exercising, etc. Each target behavior present was coded once per app; one app could contain several target behaviors. Behavior change content was coded by a trained investigator (LSdP) using the BCT taxonomy [15]. This taxonomy represents a consensus of hierarchically structured techniques developed to specify behavioral interventions. Each BCT present was coded once for each app; one app could contain multiple BCTs. A second trained coder (ALD) evaluated a subset of 8 selected apps out of 44 (18%); interrater reliability was computed with bias-adjusted kappa.

### Understandability and Actionability Assessment

Material is understandable and actionable when users of different health literacy levels can “process and explain key messages” and “identify what they can do based on the information presented” [20]. Understandability and actionability levels were evaluated using the PEMAT-A/V [20], which is a systematic method to evaluate understandability and actionability of patient education materials. It includes 13 items in five topics—Content, Word Choice & Style, Organization, Layout & Design, and Use of Visual Aids—to evaluate understandability (eg, “The material makes its purpose completely evident,” “The material uses common, everyday language,” etc) and four items for actionability (eg, “The material clearly identifies at least one action the user can take”). Each item was rated with 0 (If Disagree) or 1 (If Agree), while items that were not applicable received N/A. Item scores were added and divided by the maximum score possible, excluding items that were not applicable, and the result multiplied by 100 to get a percentage score. A second coder assessed understandability and actionability scores for the same subset of apps used for BCT coding; interrater reliability was computed using the intraclass correlation coefficient.

### Data Analysis

Coding was done using Microsoft Excel and all statistical analyses were performed using RStudio, version 1.1.383. We examined app characteristics, behavioral content, and PEMAT-A/V scores via descriptive statistics. We performed nonparametric tests to compare groups and to investigate the correlation between the variables of interest, given their distribution properties. Associations between behavioral content and PEMAT-A/V scores were investigated via bivariate correlations (the Spearman rank correlation coefficient, ρ). Additional exploratory analyses regarding relationships between app characteristics and these content and format properties are reported in Multimedia Appendices 3-6 for interested readers.

## Results

### Search

Of the total 704 unique apps identified in the Google Play store, 167 (23.7%) had descriptions in languages other than French; 104 apps (14.8%) were considered as targeting chronic conditions in general and 50 apps (7.1%) focused on chronic conditions in the four groups of diseases included in this review. Other chronic conditions, not considered in this work, were back pain, migraine and headache, sleep apnea, and depression, among others. For app selection, agreement between reviewers was substantial (κ=.62). Reconciliation was done by a third reviewer when agreement was not reached after discussion between the first two. A total of 50 apps out of 704 (7.1%) met the inclusion criteria. Nonetheless, 3 of the selected apps required other connected objects (eg, connected glucometer, blood glucose sensor, or smart watch), 3 had descriptions in French but app content was entirely in English, and 2 pairs of apps had different names but the same content, so 1 app from each pair was removed; the number of excluded apps was 8. When the second round of coding of BCT content and understandability and actionability scores was performed in October 2018 for calculation of interrater reliability and score revision, 3 apps were no longer available on the marketplace and were removed from the analysis.

**Figure 1:**
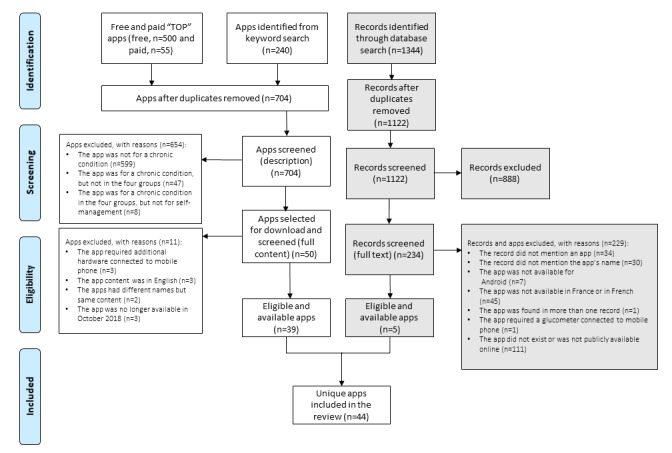
Flowchart of the screening process.

The literature search yielded 1344 abstracts, and 234 manuscripts were assessed based on full text. The kappa indicating interrater reliability was .33 (ie, fair agreement), and a third reviewer did reconciliation by checking all records disagreed upon by the first two reviewers. Many of the manuscripts found through the literature search were reviews, as well as reviews of reviews (n=282). No peer-reviewed or conference papers from French institutions were selected for full-text screening. A total of 7 [28-34] out of 234 (3.0%) manuscripts selected mentioned at least one native app for Android available in France and in French; these were included in our review. Of the 7 apps, one was present in two different articles and one required a glucometer connected to the mobile phone, which prevented app use. This resulted in 5 distinct apps downloaded from the Google Play store. Apps found through the literature search were not present in the list of apps extracted previously from the Google Play store. Finally, we analyzed a list of 44 unique native apps: 5 from the literature search and 39 from the marketplace search (see Figure 1).

### Sample Characteristics of Apps

A total of 44 apps were downloaded and analyzed, most of them targeting diabetes (19/44, 43%). The least-represented category was cancer (1/44, 2%). Table 1 shows the main characteristics of the apps analyzed.

**Table 1:** Characteristics of apps sample.

Characteristics		Apps (N=44)
**Disease category, n (%)**		
	Cancers	1 (2)
	Respiratory diseases	6 (14)
	Cardiovascular diseases	10 (23)
	Diabetes	19 (43)
	Other	8 (18)
Number of stars, mean (SD)		4.18 (0.48)
Number of stars, range		3-5
Number of user ratings, mean (SD)		16,140.1 (61,401.6)
Number of user ratings, range		2-374,462
**Downloads, n (%)**		
	50+	1 (2)
	100+	3 (7)
	500+	4 (9)
	1000+	4 (9)
	5000+	4 (9)
	10,000+	6 (14)
	50,000+	3 (7)
	100,000+	10 (23)
	500,000+	3 (7)
	1,000,000+	3 (7)
	5,000,000+	2 (5)
	10,000,000+	1 (2)
**Gratuity, n (%)**		
	Paid app	1 (2)
	With paid features	14 (32)
	Without paid features	29 (66)
**Developer, n (%)**		
	Nonprivate organization^a^	4 (9)
	Pharmaceutical or medical device company	13 (30)
	Private app-development company	27 (61)

^a^Nongovernmental organizations, public institutions, or European projects.

### Target Behavior and Behavior Change Technique Characteristics

We identified the presence of 10 target behaviors and 21 BCTs in our app sample (see Table 2). The maximum number of target behaviors observed per app was 7 in 2 apps; the median number was 2. A total of 5 apps did not present any target behaviors. A total of 4 apps had no BCTs present and another 4 had only 1 BCT, while only 1 app had more than 10 (n=12). The median number of BCTs per app was 3 (range 0-12).

The kappa for interrater reliability in BCT coding was .68 (ie, substantial agreement); the reconciliation process was used to revise coding in the whole app sample.

**Table 2:** Prevalence of target behaviors and behavior change techniques (BCTs) in the app sample.

Target behaviors and BCTs		Occurrence in apps (N=44), n (%)
**Target behavior**		
	Tracking symptoms	26 (59)
	Medication adherence	13 (30)
	Tracking diet	12 (27)
	Tracking weight	11 (25)
	Archiving health information	9 (20)
	Physical activity	6 (14)
	Attending medical appointments	3 (7)
	Tracking emotional symptoms	3 (7)
	Drinking water	2 (5)
	Tracking sleep	2 (5)
**BCT**		
	Self-monitoring of outcome(s) of behavior	31 (70)
	Feedback on outcome(s) of behavior	25 (57)
	Self-monitoring of behavior	19 (43)
	Prompts and cues	17 (39)
	Information about health consequences	14 (32)
	Goal setting (outcome)	11 (25)
	Graded tasks	5 (11)
	Action planning	4 (9)
	Biofeedback	3 (7)
	Feedback on behavior	3 (7)
	Social support (practical)	2 (5)
	Social comparison	2 (5)
	Instruction on how to perform the behavior	1 (2)
	Demonstration of the behavior	1 (2)
	Credible source	1 (2)
	Monitoring of emotional consequences	1 (2)
	Social reward	1 (2)
	Goal setting (behavior)	1 (2)
	Social support (unspecified)	1 (2)
	Social support (emotional)	1 (2)

The most common target behaviors were *tracking symptoms* (eg, in the case of apps for hypertension, these included measuring blood pressure and recording the values in the app journal) (26/44, 59%); *medication adherence* (eg, recording medication name and dosage and setting alarms to remember taking them) (13/44, 30%); *tracking diet* (eg, in the case of diabetes apps, these included noting food quantities in an app journal) (12/44, 27%); *tracking weight* (11/44, 25%); and *archiving health information* (eg, recording clinical test results in an app journal) (9/44, 20%). For BCTs, the most common were *self-monitoring of outcome(s) of behavior* (31/44, 70%), followed by *feedback on outcome(s) of behavior* (25/44, 57%), *self-monitoring of behavior* (19/44, 43%), *prompts and cues* (17/44, 39%), *information about health consequences* (14/44, 32%), and *goal setting (outcome)* (11/44, 25%). All target behaviors and BCTs mentioned above were encountered in more than 20% of analyzed apps.

**Figure 2:**
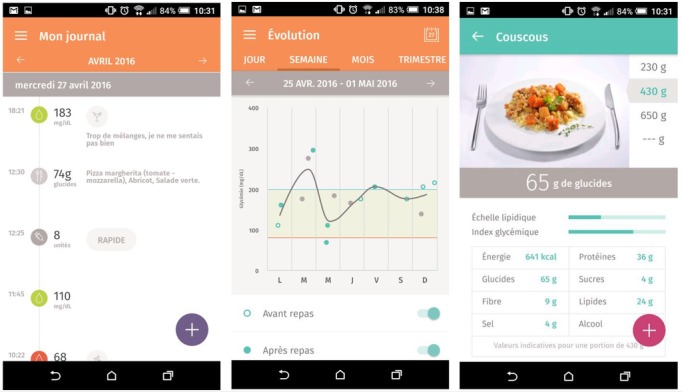
Screenshots from the Gluci-Check app from the Google Play store. The screenshots show examples of target behaviors and behavior change techniques (BCTs) used in apps. The left-hand screenshot corresponds to the target behavior *tracking symptoms* and the BCT *self-monitoring of outcome(s) of behavior*; the middle screenshot corresponds to the BCTs *feedback on outcome(s) of behavior* and *goal setting (outcome)*; the right-hand screenshot corresponds to the target behavior *tracking diet* and the BCT *self-monitoring of behavior*.

Figure 2 shows examples of target behaviors and BCTs. The left-hand screenshot shows a blood glucose journal, corresponding to the target behavior *tracking symptoms* and the BCT *self-monitoring of outcome(s) of behavior*. The middle screenshot shows a graph with blood glucose level variation through a period of one week along with the blood glucose target range defined by the user and his or her health care provider, corresponding to the BCTs *feedback on outcome(s) of behavior* and *goal setting (outcome)*. The right-hand screenshot shows a food journal corresponding to the target behavior *tracking diet* and the BCT *self-monitoring of behavior*. This specific app is designed for people with type 1 diabetes and knowing the amount of carbohydrates in meals is essential for adjusting insulin dosage.

### Understandability and Actionability Scores

**Figure 3:**
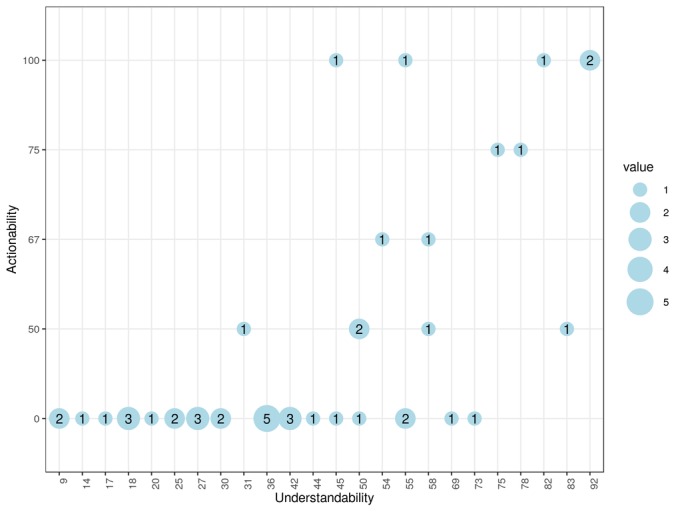
Understandability versus actionability; the circle size and label indicate the number of apps with the corresponding two scores.

Out of the 44 apps, 2 (5%) had an actionability score of 100%. The mean understandability score was 43.50% (SD 22.24), the median was 42% (interquartile range [IQR] 28), and the values ranged from 9% to 92%. For actionability, the mean score was 23.50% (SD 36.86), the median was 0% (IQR 50), and values ranged from 0% to 100%. A total of 30 apps out of 44 (68%) had null actionability (ie, they had no clearly stated actions the user could take regarding the self-management behaviors the app targeted). Figure 3 shows the co-occurrence of understandability and actionability scores in the sample.

The kappa for interrater reliability for understandability scores was .65 (ie, substantial agreement) and for actionability scores was .02 (ie, poor agreement). Both coders rated actionability as low for most apps. Differences were mostly related to an interpretation ambiguity in the first item of the actionability assessment—“The material clearly identifies at least one action the user can take”—upon which all three other actionability items were dependent. The fact that there were only four items to evaluate also influenced the different scores. Disagreements were discussed and the process of reconciliation led to revising the scores for the other apps in the sample.

Understandability and actionability scores were positively correlated (ρ=.67, *P*<.001) and so were the number of BCTs and target behaviors per app (ρ=.62, *P*<.001). Understandability had a positive correlation to the number of BCTs per app (ρ=.35, *P*=.02) and number of target behaviors per app (ρ=.31, *P*=.04). This may suggest that apps with more target behaviors and BCTs also tended to present this content in a way that is easier to understand. Actionability had moderate positive correlations to target behaviors per app (ρ=.24, *P*=.12) and BCTs per app (ρ=.29, *P*=.054), which were not statistically significant.

## Discussion

### Principal Findings

The use of mHealth apps for supporting health-related behavior change and patient empowerment is being widely discussed as a solution to health care challenges worldwide, particularly for chronic conditions. This review showed that in 2018 in France, the potential of mHealth is far from being achieved. The apps available for download on Google Play had relatively limited behavior change content and, although moderately easy to understand for diverse audiences, they did not commonly point to clear actions users may take to self-manage the condition targeted. To better support patients with chronic conditions, apps can be improved by building on more solid behavior change research and applying it in ways that are easier to understand and act upon.

By searching directly on Google Play, among 704 apps with different purposes and languages, we found only 39 apps available in French targeting self-management in the four groups of chronic conditions with the highest mortality rates—cardiovascular diseases, cancers, respiratory diseases, and diabetes. This suggests that, for a patient or health care professional interested in using apps to manage a chronic condition, finding an appropriate app can be challenging. Moreover, our literature search identified only 5 apps available in France and in French. In another review with similar methodology performed in Canada with mHealth apps in English, of the total of 107 apps for depression analyzed, 48 were found through a literature search [24]. This highlights the variability of mHealth apps offerings worldwide, in terms of both availability and research, and the importance of studying the evolution of this domain in different countries and languages.

Targeting specific key behaviors in interventions and combining active components (ie, BCTs) that are potentially effective for behavior change in the context of chronic conditions is crucial to achieve the intended change and, consequently, improve and maintain quality of life [35,36]. In our sample, the most common target behavior was *Tracking symptoms*, and an important prevention behavior, *Physical activity*, was present in only 6 apps. The median number of target behaviors per app was 2, ranging from 0 to 5. The modifiable behaviors described as priorities by the World Health Organization as risk factors increasing mortality rates, such as tobacco use, alcohol consumption, and excessive salt intake, were not addressed in any of the analyzed apps. There was substantial variation in the number of BCTs present and the majority of the apps focused on self-monitoring, confirming the focus on monitoring behaviors as previously shown in the literature. We observed 20 BCTs in our sample and the median number of BCTs per app was 3, ranging from 0 to 12. Conroy et al [22] observed 26 BCTs in total and the most top-ranked apps for physical activity incorporated less than 4 BCTs, ranging from 1 to 13. Direito et al [23] found 26 BCTs in total, with an average of 8.1 BCTs per app, ranging from 2 to 18; free apps, such as most of the apps evaluated in our study, presented a slightly smaller average (ie, 6.6, ranging from 3 to 14). In both studies, *provide instruction* was the most common BCT, which was not observed in the case of chronic condition mHealth apps in our study. Similar to the results of Martinez-Pérez et al [37] regarding mHealth apps for the most prevalent conditions by the World Health Organization, we found more assistive and monitoring characteristics in the apps in our sample than informative and educational ones, also reinforced by the most common target behavior *tracking symptoms*. *Demonstration of the behavior* was an uncommon BCT, and videos or illustrations to clarify the use of measurement equipment, such as blood glucose or pressure monitors, were rare. Furthermore, only a third of apps presented *information about health consequences*, which is indispensable for understanding complications related to the chronic conditions discussed here. *Goal setting (outcome)* was also frequent (>60%) in physical activity apps [22], while in our work, it was present in less than one-fourth of apps. Goal management BCTs (ie, *goal setting* and *goal review*) were found to be effective in physical activity and dietary behavior change interventions [38-41]. Also, the combination of self-monitoring techniques with at least one other self-regulation technique (ie, intention formation, feedback on performance, specific goal setting, and review of behavioral goals) is shown to be more effective than other interventions [40]. Moreover, *action planning*, which is highly related to actionability and overcoming emergencies, was present in only 5 apps. We were thus able to identify limited behavioral change content and a focus on monitoring rather than goal management or education.

The apps in our study were to a large extent not suitable for low literacy audiences. The median understandability and actionability scores were 42% and 0%, respectively. In a previous study that applied the PEMAT-A/V to 43 apps intended for parent education (ie, parenting, child health, or infant health) [42], 30 apps had understandability scores between 76% and 100%, while for actionability, 19 apps had scores in this range. We found most apps had hard-to-read text (ie, small font and too much text). In previous work with a different methodology, Meppelink et al [43] showed that almost 80% of Dutch health information websites were over the recommended B1 reading level: B1 reading level means 95% of the population can understand the information. In addition, the predominantly low actionability of the apps in our study shows that we are still far from fulfilling the potential of mHealth tools to increase patient autonomy. More than half of the analyzed apps did not present any clearly stated action and they did not have any suggestions concerning the data recorded by users on health-related events. For mHealth apps to fully achieve their potential to support chronic condition treatment, clearly indicating actions is imperative (eg, diabetes apps need to indicate that patients need to intervene immediately if high or low blood glucose is recorded and give concrete physical activity suggestions to users). In our study, apps with more target behaviors and BCTs were also more understandable, indicating that developers who consider behavioral content may also be more careful with making sure apps are comprehensible for users; levels of actionability were low irrespective of behavioral content. We therefore highlight actionability as a priority to address in app development: stating actions users can take, addressing users directly when describing actions, presenting actions in short explicit steps, and explaining how to use data visualization to take action [20].

### Strengths and Limitations

First, our study used a three-pronged search strategy to identify apps relevant for our research questions: two strategies likely employed by users to identify apps in the marketplace (ie, top-ranked mHealth apps and active keyword search) and one strategy to identify apps that have been subject to scientific research (ie, a literature search). However, only the Android app marketplace was examined in this study and, although Google has the largest portion of the mobile app market and most apps are present in both marketplaces, not considering the second-most popular app marketplace (ie, Apple App Store) can lead to omission of relevant apps. Nonetheless, we believe our search strategies enabled us to obtain a representative sample to describe the current state-of-the-art in mHealth self-management support. Second, we only considered peer-reviewed papers and conference articles published in English, even though we were looking for apps available in France and in French. A future study of the iOS marketplace and French databases may be useful to complement our findings. We have also downloaded and assessed both behavioral content as well as understandability and actionability by two independent coders interacting directly with the apps, not only the descriptions available in the commercial marketplace. Thus, we were able to obtain a comprehensive assessment of the properties examined and reflect also on the assessment tools used.

We identified several issues for further improvement. First, while the BCT taxonomy enables systematic coding with good intercoder reliability, there is no consensus to date on classifying target behaviors apart from broad domains [44]. We have followed commonly used terminology to describe target behaviors in this study, yet our descriptions would have certainly benefited from standardized labels. Target behavior definition and selection is a key step in behavior change [45] and working toward a consensus on target behavior classification would further facilitate evidence synthesis. Second, since PEMAT-A/V was developed for educational materials using audio and video resources, we encountered a few difficulties when applying its criteria to apps. For example, app names and descriptions are commonly less informative in apps than what is expected for other health-related educational materials, names may be unrelated to the condition, and descriptions do not necessarily contain all app features. These characteristics may be interpreted as low understandability but may also be due to different design conventions in apps, which may have to be considered as an underestimation of understandability in our sample. PEMAT-A/V was selected after careful review of several tools, as it was considered best suited to app assessment by the research team. However, we would support a future adaptation of PEMAT-A/V for apps, which could aim to reconcile the usual brevity of the app medium with the requirements of effective communication for different audiences. Third, our study focused on the content and format of apps and excluded other criteria for judging app quality, from user engagement and functionality [46] to data security and ethical and legal standards [47]. A comprehensive evaluation was beyond the scope of our review and would need to consider multiple dimensions.

### Conclusions

Our findings suggest that mHealth apps available in France could be improved in terms of content and format. They also illustrate how two readily available tools—the BCT taxonomy and the PEMAT-A/V—can provide useful insights into the potential of an app to support patient empowerment. These tools can be used by different stakeholders in app development or to assess the existing offerings to ensure an effective contribution of apps to patient care; we would recommend their inclusion in broader app development and evaluation guidelines. Given the prevalence of the chronic conditions considered here, it is essential to make sure different levels of health literacy are considered when developing health-related materials. Also, the development of mHealth apps should involve users and consider their behavioral support needs and be accompanied by research on whether their content and use are able to effectively change behavior. Apps could also benefit from integrating more instructions for intended users on actions to perform, more modifiable behavioral risk factors, and more behavior change content, especially BCTs associated with increased effectiveness in modifying target behaviors.

## Appendix

Multimedia Appendix 1 Preferred Reporting Items for Systematic Reviews and Meta-Analyses (PRISMA) checklist.

Multimedia Appendix 2 Search strategy in the Google Play store and in PubMed (ie, MEDLINE), IEEE, and Web of Science databases.

Multimedia Appendix 3 App sample with data extracted from the Google Play store.

Multimedia Appendix 4 Dataset used to perform analyses.

Multimedia Appendix 5 R markdown script.

Multimedia Appendix 6 R markdown report with descriptive statistics and inferential statistical and exploratory analyses.

## Abbreviations

BCT: behavior change technique
IQR: interquartile range
mHealth: mobile health
PEMAT-A/V: Patient Education Material Assessment Tool for audiovisual materials
PRISMA-P: Preferred Reporting Items for Systematic Reviews and Meta-Analyses Protocols
PROSPERO: International Prospective Register of Systematic Reviews
