# Supplementary material for: Behavior Change Content, Understandability, and Actionability of Chronic Condition Self-Management Apps Available in France: Systematic Search and Evaluation
Source: JMIR Mhealth Uhealth. 2019 Aug 26;7(8):e13494. doi: 10.2196/13494 (PMC6735304; doi:10.2196/13494)
Supplement: Multimedia Appendix 2 [file mhealth_v7i8e13494_app2.pdf]

Google Play store search

500 first “TOP” apps in “Médecine” category +

55 available first “TOP” paid apps in “Médecine” category +

20 first apps found for each keyword (total of 240 apps) as shown in the table below:

Category	Keywords
Cardiovascular diseases	maladie cardiaque, maladie coeur, AVC accident vasculaire cérébral, infarctus
Respiratory diseases	maladie pulmon, asthme, BPCO, maladie respiratoire,
Diabetes	diabète, diabète type 1, diabète type 2
Cancer	cancer

Literature Search

Category	Medical Subject headings (MeSH)	Keywords
Chronic conditions	Cardiovascular Diseases, Respiratory Tract Diseases, Neoplasms, Diabetes Mellitus, Asthma	diabet* OR cardiovascular OR cardiac OR myocardial OR coronary OR heart OR stroke OR cerebrovascular OR vascular OR asthma* OR respiratory OR lung OR cystic fibrosis OR pulmonary OR cancer OR cancers OR carcinoma OR carcinomas OR tumor OR tumors OR tumour OR tumous OR malignant OR malignancy OR malignancies OR leukemia OR lymphoma
Self-management	Self-management, Self Care	Self-management OR selfmanag* OR self-care OR selfcar*

mHealth apps		mobile OR smartphone* OR android* OR app OR apps OR iphone* OR ipad* OR m-health

Search strategies:

PubMed

("Cardiovascular Diseases"[Mesh] OR "Respiratory Tract Diseases"[Mesh] OR "Neoplasms"[Mesh] OR "Diabetes Mellitus"[Mesh] OR diabet*[tw] OR "cardiovascular"[tw] OR "cardiac"[tw] OR "myocardial"[tw] OR "coronary"[tw] OR "heart"[tw] OR "stroke"[tw] OR "cerebrovascular"[tw] OR "vascular"[tw] OR "Asthma"[Mesh] OR asthma*[tw] OR "respiratory"[tw] OR "lung"[tw] OR "cystic fibrosis"[tw] OR "pulmonary"[tw] OR "cancer"[tw] OR "cancers"[tw] OR "carcinoma"[tw] OR "carcinomas"[tw] OR "tumor"[tw] OR "tumors"[tw] OR "tumour"[tw] OR "tumous"[tw] OR "malignant"[tw] OR "malignancy"[tw] OR "malignancies"[tw] OR "leukemia"[tw] OR "lymphoma"[tw]) AND

("Self-Management"[Mesh] OR "self management"[tw] OR selfmanag*[tw] OR "Self Care"[Mesh] OR "self care"[tw] OR selfcar*[tw]) AND

(mobile[tw] OR smartphone*[tw] OR android*[tw] OR app[tw] OR apps[tw] OR iphone*[tw] OR ipad*[tw] OR m-health[tw]) AND ("2012"[Date - Create] : "3000"[Date - Create]) AND (("2012/01/01"[PDat] : "3000/12/31"[PDat]))

IEEE

((((("smart phone" OR mobile* OR personal computing OR app) AND (self-management OR self-care OR self manag* OR self car*)) AND ((cardiovascular diseases OR cardiac disease OR heart disease) OR diabetes OR (cancer OR myeloma OR lymphedema) OR (pulmonary* OR asthma)))))

WoS

#1 - TS=(mHealth OR mobile* OR smart phone OR app OR android)

#2 - TS=(self-management OR self-care OR self manag* OR self car*)

#3 - #1 AND #2

#4 - TS=("cardiovascular diseases" OR heart diseases OR vascular diseases OR heart attack OR heart failure)

#5 - TS=("diabetes mellitus" OR diabetes OR type 1 diabetes OR type 2 diabetes)

#6 - TS=(cancer OR neoplasm OR lymphoma OR leukemia)

#7 - TS=("respiratory tract diseases" OR "pulmonary diseases" OR asthma OR "chronic obstructive pulmonary disease")

#8 - #4 OR #5 OR #6 OR #7

#9 - #3 AND #8
