# Supplementary material for: Behavior Change Content, Understandability, and Actionability of Chronic Condition Self-Management Apps Available in France: Systematic Search and Evaluation
Source: JMIR Mhealth Uhealth. 2019 Aug 26;7(8):e13494. doi: 10.2196/13494 (PMC6735304; doi:10.2196/13494)
Supplement: Multimedia Appendix 6 [file mhealth_v7i8e13494_app6.pdf]

BCT, understandability and actionability in mHealth self-management apps for chronic conditions available in France

2019-06-18

Summary

This document presents a summary of the data analysis performed for the paper entitled “Behaviour change content, understandability and actionability in mHealth self-management apps for chronic conditions available in France: a systematic review”, based on data collected on mHealth self-management apps selected in March-April 2018 in France.

First, we performed descriptive statistics of app characteristics, presence of target behaviours and BCTs, and understandability and actionability scores. Second, we presented statistical analysis of the variables of interest, with normality tests and appropriated statistical tests to study the relationship between them.

Descriptive statistics

Sample characteristics

The sample included 44 apps. Behaviour Change Techniques (BCT) were computed for each app, along with Target Behaviour (TB) and PEMAT scores for Understandability and Actionability. Characteristics such as stars, ratings, number of downloads, type of developer, version and last update were also extracted from the marketplace and are present in the dataset. Table 1 presents descriptive statistics of app characteristics.

Table 1: Descriptives of app characteristics

Indicator	Levels	Statistics
n		44
Stars (mean (SD))		4.18 (0.49)
Ratings (mean (SD))		16140.09 (61401.60)
Downloads (%)	50	1 (2.3)
	100	3 (6.8)
	500	4 (9.1)
	1000	4 (9.1)
	5000	4 (9.1)
	10000	6 (13.6)
	50000	3 (6.8)
	100000	10 (22.7)
	500000	3 (6.8)
	1000000	3 (6.8)
	5000000	2 (4.5)
	10000000	1 (2.3)
Category (%)	Cancer	1 (2.3)
	Cardiovascular Diseases	10 (22.7)
	Diabetes	19 (43.2)
	Other	8 (18.2)
	Respiratory Diseases	6 (13.6)
Developer (%)	Non-private	4 (9.1)

Indicator	Levels	Statistics
	Pharma/Medtech	13 (29.5)
	Private company	27 (61.4)

Behavioural content

Target behaviours (TB)

The number and percentage of apps in which each target behaviour was identified in this sample are presented in Table 2. The distribution of the total number of TBs per app is presented in Figure 1 as a histogram.

Table 2: TB occurences in app sample

Target Behaviour	Total	Percentage
Tracking symptoms	30	68.18
Medication adherence	13	29.55
Tracking diet	12	27.27
Tracking weight	11	25.00
Archiving health information	9	20.45
Physical activity	6	13.64
Attending medical appointments	3	6.82
Tracking emotional symptoms	3	6.82
Drinking water	2	4.55
Tracking sleep	2	4.55

Interpretation

We observed a total of 10 TBs. More than a third of the apps (n = 17, 38.64%) targeted none or only one behaviour. Median number of TB per app was 2, ranging from 0 to 7.

Behaviour Change Techniques (BCTs)

The number and percentage of apps in which each BCT was identified are presented in Table 3 for BCTs with at least one occurrence in this sample. Figure 2 presents a histogram of the total number of BCTs per app.

Table 3: BCT occurrence in app sample

Behaviour Change Technique	Total	Percentage
2.4 Self-monitoring of outcome(s) of behaviour	31	70.45
2.7 Feedback on outcome(s) of behaviour	25	56.82
2.3.Self-monitoring of behaviour	19	43.18
7.1 Prompts and cues	17	38.64
5.1 Information about health consequences	14	31.82
1.3 Goal setting (outcome)	11	25.00
8.7 Graded tasks	5	11.36
1.4 Action planning	4	9.09
2.6 Biofeedback	3	6.82
2.2 Feedback on behaviour	3	6.82
3.2 Social support (practical)	2	4.55
6.2 Social comparison	2	4.55
4.1 Instruction on how to perform the behaviour	1	2.27
6.1 Demonstration of the behaviour	1	2.27

Behaviour Change Technique	Total	Percentage
9.1 Credible source	1	2.27
5.4 Monitoring of emotional consequences	1	2.27
10.4 Social reward	1	2.27
1.1 Goal setting (behaviour)	1	2.27
3.1 Social support (unspecified)	1	2.27
3.3 Social support (emotional)	1	2.27

Interpretation

We observed a total of 20 BCTs. Nearly one fifth of the apps (n = 8, 18.18%) had none or only one BCT present. Median number of BCT per app was 3, ranging from 0 to 12.

PEMAT

Histograms with Understandability and Actionability scores are presented in Figure 3 and Figure 4, respectively. Figure 5 shows the co-occurrence of Understandability (x-axis) and Actionability (y-axis) scores in this sample (circle size and label indicate the number of apps with the corresponding 2 scores).

Interpretation

Overall, Understandability scores were higher than Actionability scores. In the sample, 30 apps had null Actionability scores, while 0 had null Understandability scores. On the other hand, 5 apps had scores of 100% Actionability and the maximum score of Understandability was 92. Plot shows that apps with higher Actionability scores also had higher Understandability scores. We also performed a Wilcoxon rank sum test to examine this difference and results show that Understandability values for apps with non-null Actionability (mean = 64.5) and with null Actionability (mean = 33.7) were significantly different (p = 0.00002).

Inferential statistics

This section includes bivariate and multivariate analysis to compare and examine the relationship between computed variables (TB per app, BCT per app, Understandability and Actionability) and apps characteristics extracted from Google Play store (Stars, Ratings, Downloads and Sales on app).

Normality tests were performed on quantitative variables to choose parametric or non-parametric statistical tests (presented below). Stars, BCT per app, TB per app and Actionability are not normal, while Ratings and Understandability are. Therefore, Wilcoxon rank sum test was chosen to perform bivariate analysis and Kruskal-Wallis test to perform multivariate analysis. We also used the Spearman's coefficient (rho) to compute correlations.

```
## $Stars
##
##  Shapiro-Wilk normality test
##
## data:  newX[, i]
## W = 0.96183, p-value = 0.1523
##
##
## $Ratings
##
##  Shapiro-Wilk normality test
##
## data:  newX[, i]
```

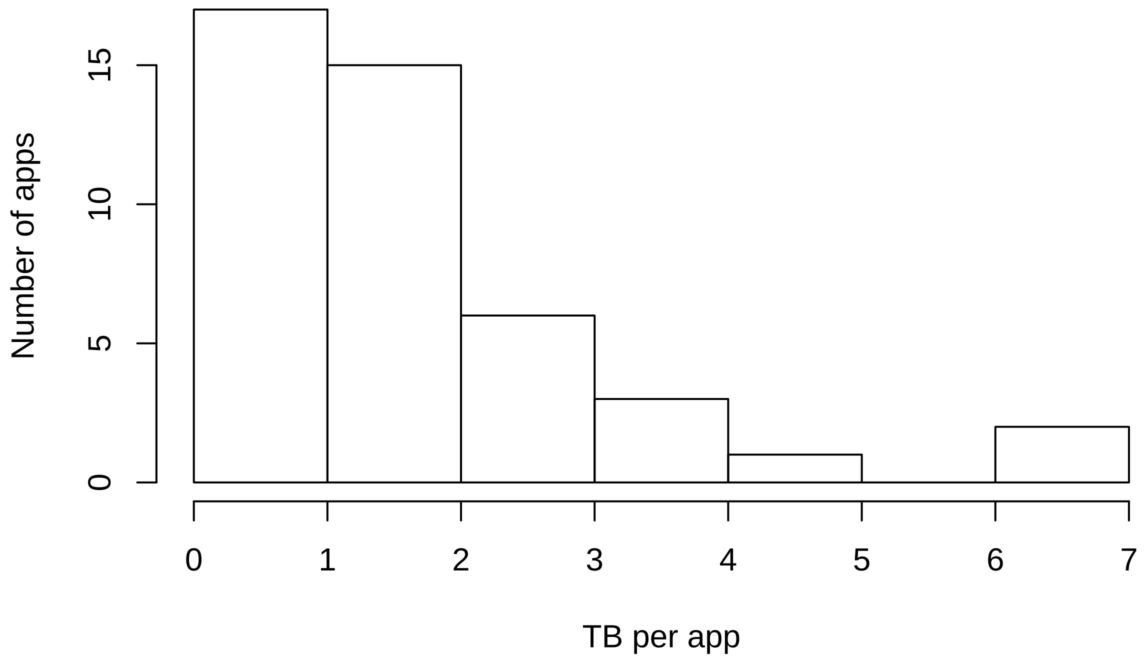

Figure 1: Figure 1: Histogram TB per app

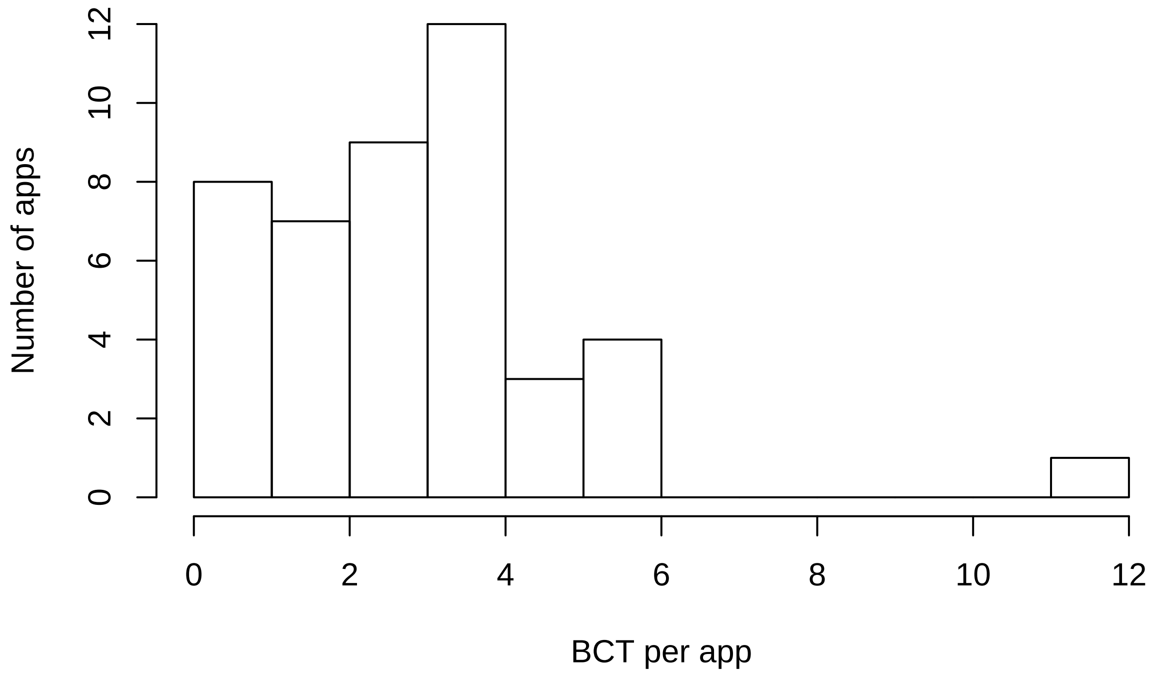

Figure 2: Figure 2: Histogram BCT per app

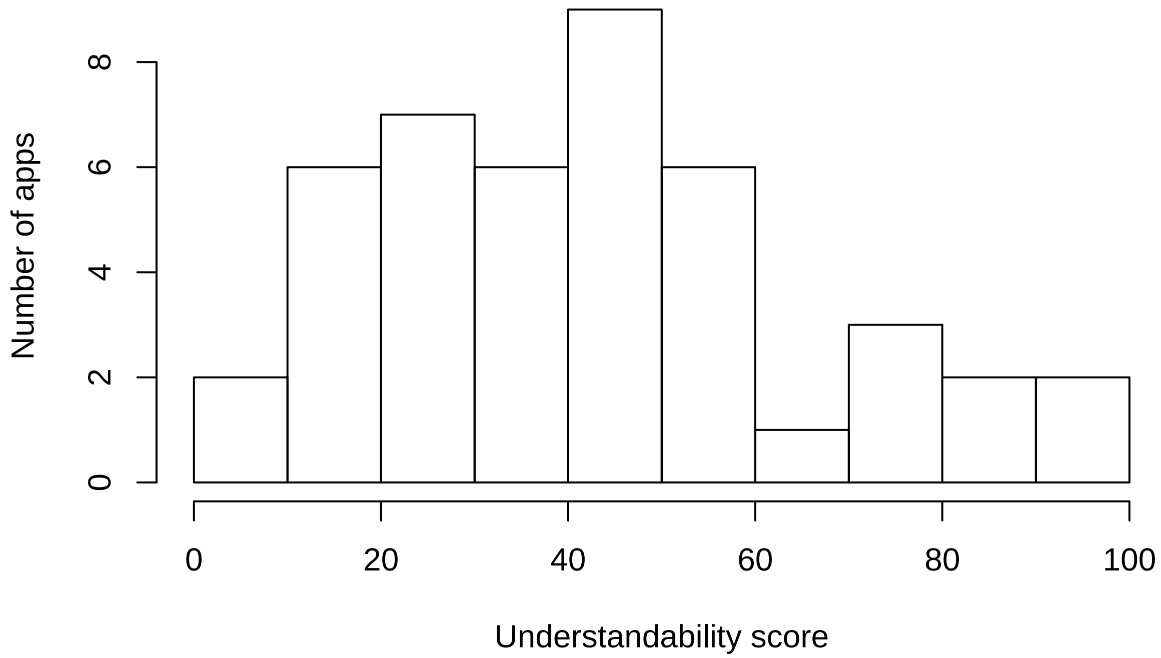

Figure 3: Figure 3: Histogram with Understandability scores

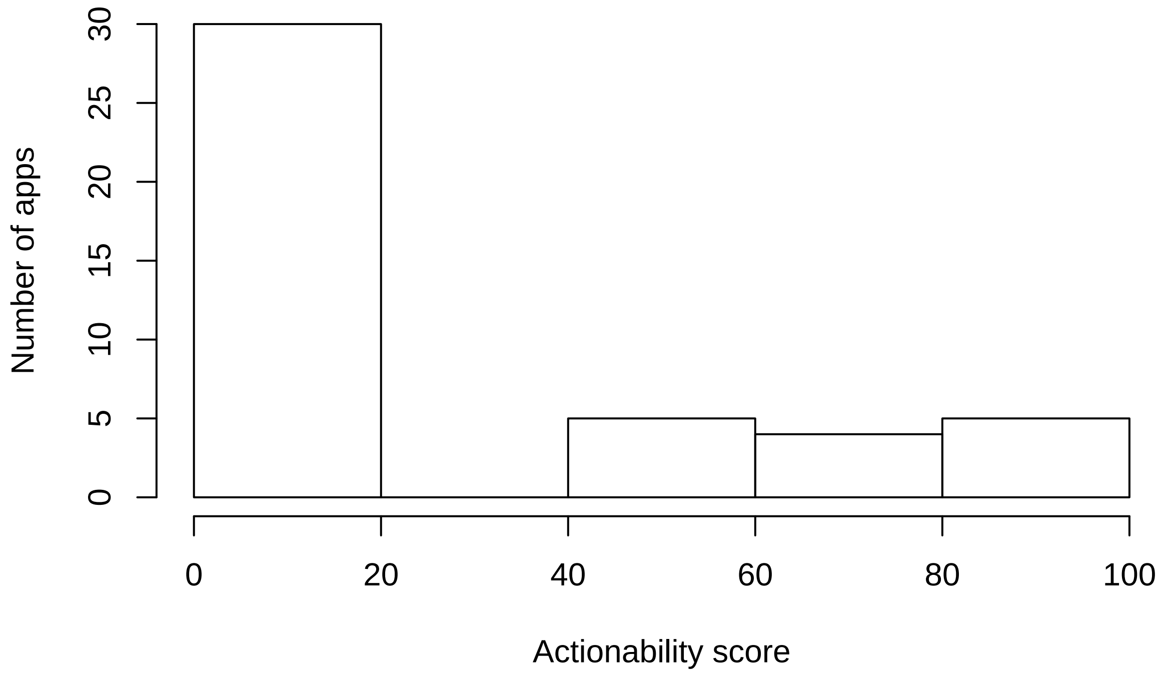

Figure 4: Figure 4: Histogram with Actionability scores

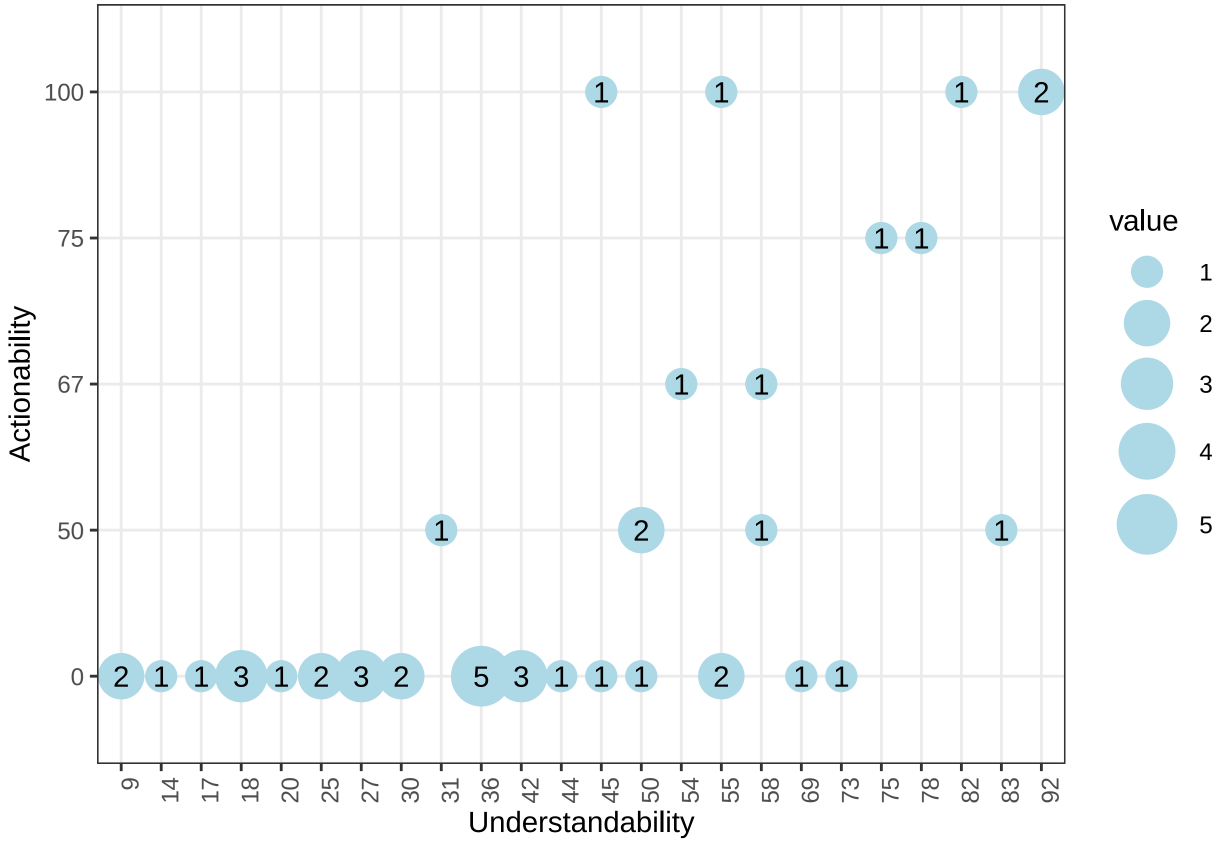

Figure 5: Figure 5: Understandability versus Actionability

```
## W = 0.2834, p-value = 2.359e-13
##
##
## $`BCT per app`
##
##  Shapiro-Wilk normality test
##
## data:  newX[, i]
## W = 0.87745, p-value = 0.0002366
##
##
## $`TB per app`
##
##  Shapiro-Wilk normality test
##
## data:  newX[, i]
## W = 0.85386, p-value = 5.486e-05
##
##
## $Understandability
##
##  Shapiro-Wilk normality test
##
## data:  newX[, i]
## W = 0.95251, p-value = 0.06823
##
##
## $Actionability
##
##  Shapiro-Wilk normality test
##
## data:  newX[, i]
## W = 0.65257, p-value = 5.958e-09
```

Correlation between computed variables and ranking characteristics

Table 4 presents Spearman's correlation coefficient (rho) between ranking characteristics (Stars and Ratings) and computed variables (TB per app, BCT per app, Understandability and Actionability). Figure 4 is heatmap to illustrate these correlations.

Table 4: Correlation (Spearman's rho) between variables and ranking characteristics

	Stars	Ratings	BCT per app	TB per app	Understandability
Ratings	-0.16				
BCT per app	-0.21	0.22			
TB per app	-0.28	0.15	0.62****		
Understandability	-0.10	-0.06	0.35*	0.31*	
Actionability	-0.11	-0.20	0.29	0.24	0.67****

Interpretation

TB per app and BCT per app had a significant positive correlation (rho = 0.62, p = 0.000008), as well as Understandability and Actionability (rho = 0.67, p = 0.000001) and Understandability and BCT and TB per

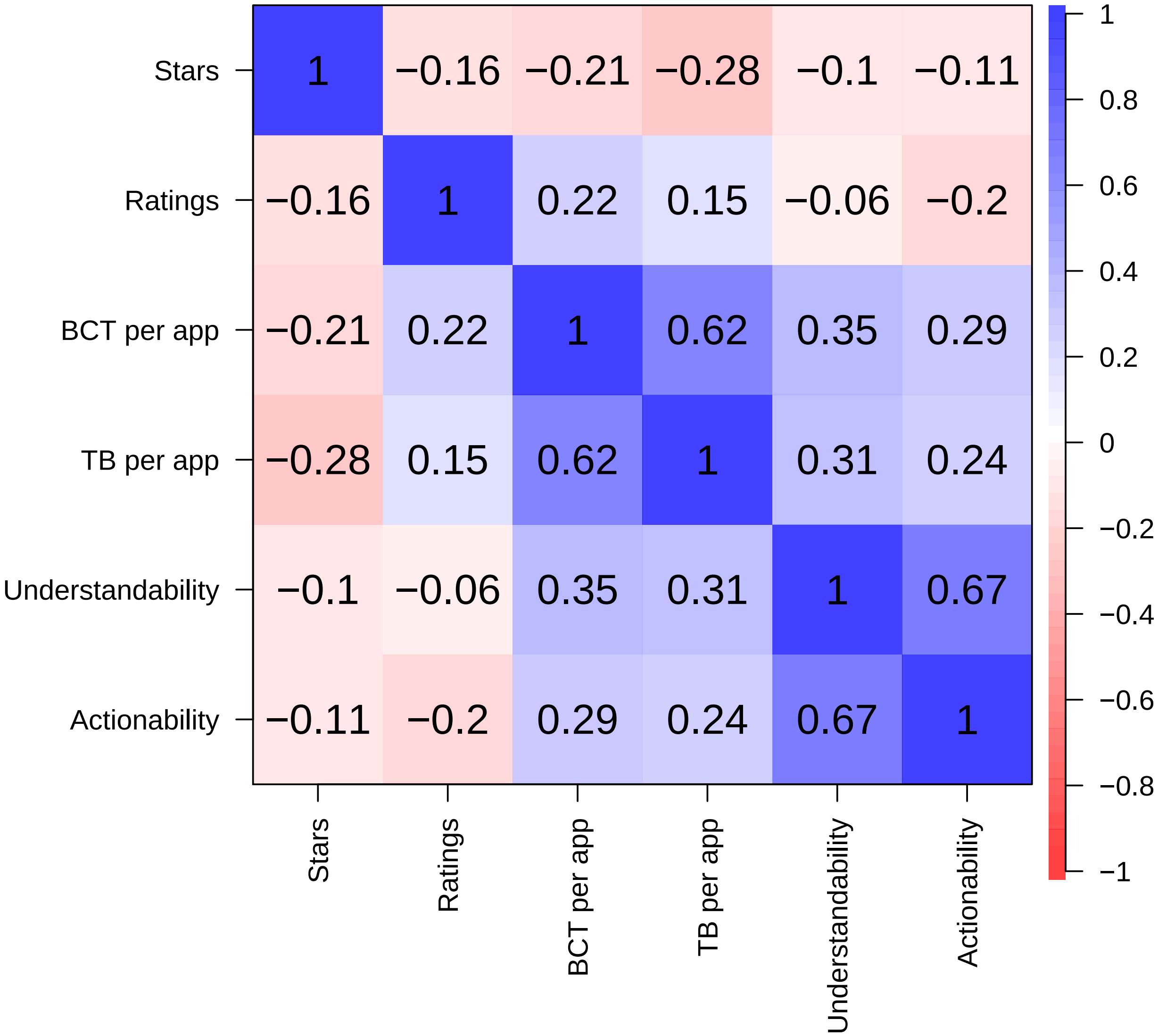

Figure 6: Figure 6: Heatmap of Spearman's correlation (rho) between variables and ranking characteristics

app (rho = 0.35, p = 0.022) and (rho = 0.31, p = 0.042), respectively. A significant negative correlation between Stars and TB per app was also observed (rho = -0.28, p = 0.061).

Are stars and ratings associated with the presence of specific BCTs?

We performed Wilcoxon rank sum tests to compare the occurence of BCTs present in more than 10% of apps and ranking characteristics. Results are presented in Table 5 and Table 6.

Table 5: Comparison between Stars mean values between apps with/without BCT and correspondent p-values

BCT	W	p
2.4 Self-monitoring of outcome(s) of behaviour	289.0	0.025
2.7 Feedback on outcome(s) of behaviour	258.5	0.626
2.3.Self-monitoring of behaviour	210.5	0.529
7.1 Prompts and cues	233.0	0.942
5.1 Information about health consequences	248.0	0.343
1.3 Goal setting (outcome)	201.0	0.605
8.7 Graded tasks	133.0	0.194

Table 6: Comparison between Ratings mean values between apps with/without BCT and correspondent p-values

BCT	W	p
2.4 Self-monitoring of outcome(s) of behaviour	107.0	0.016
2.7 Feedback on outcome(s) of behaviour	153.0	0.047
2.3.Self-monitoring of behaviour	171.5	0.121
7.1 Prompts and cues	235.5	0.895
5.1 Information about health consequences	208.0	0.970
1.3 Goal setting (outcome)	125.0	0.129
8.7 Graded tasks	99.5	0.956

Interpretation

There was significant difference in Stars mean values between apps with (mean = 4.07) and without (mean = 4.45) BCT 2.4 Self-monitoring of outcome(s) of behaviour (p = 0.025). For Ratings, there was a significant difference between apps with (mean = 22734.71) and without (mean = 414.46) BCT 2.4 Self-monitoring of outcome(s) of behaviour as well (p = 0.016), but also between apps with (mean = 25889.12) and without (mean = 3312.42) BCT 2.7 Feedback on outcome(s) of behaviour (p = 0.047).

Are there differences between BC content and PEMAT scores in apps produced by different types of developers?

Table 7 presents comparisons of computed variables (TB per app, BCT per app, Understandability and Actionability) between each category of developer (“Private company”, “Non-Private”, “Pharma/MedTech”). A paired test for Actionability scores by developer type is also presented.

Table 7: Comparison between variables mean values by developer types and correspondent p-values

Variables	statistics	pvalue
TB per app	1.48	0.478
BCT per app	2.17	0.337
Actionability	7.48	0.024

Variables	statistics	pvalue
Understandability	4.32	0.115

```
##
##  Pairwise comparisons using Wilcoxon rank sum test
##
## data:  app_data$Actionability and app_data$Developer
##
##                 Non-private Pharma/Medtech
## Pharma/Medtech  0.416       -
## Private company 0.019       0.099
##
## P value adjustment method: bonferroni
```

Interpretation

There was a significant difference in Actionability scores between "Non-private" (mean = 62.5) and "Private company" (mean = 12.67) groups (adjusted p = 0.019).

Are BC content and/or PEMAT scores related to number of downloads?

To examine the relationship between computed variables (BCT per app, TB per app, Understandability and Actionability scores) and number of downloads, we performed Kruskal-Wallis tests, as the variable Dowloads is presented in 12 ranges. Table 8 present the results; no significant difference between variables considering number of downloads was observed.

Table 8: Comparison between variables mean values by number of downloads and correspondent p-values

Variables	statistics	pvalue
TB per app	8.14	0.700
BCT per app	14.67	0.198
Actionability	15.00	0.183
Understandability	13.27	0.276

Are there differences in PEMAT scores and BC content between apps with/without sales in app?

To examine the relationship between computed variables (BCT per app, TB per app, Understandability and Actionability scores) and the presence of paid features, we also performed Kruskal-Wallis tests. Table 9 presents the results; no significant difference between variables in paid, with sales and without sales groups was observed.

Table 9: Comparison between variables mean values by presence of paid features and correspondent p-values

Variables	statistics	pvalue
TB per app	3.07	0.216
BCT per app	1.97	0.374
Actionability	2.30	0.317
Understandability	0.01	0.995

What BCTs and TBs occur together?

A Spearman's correlation heatmap between observed BCTs and TBs is presented in Figure 7.

Interpretation

This figure shows the strength of the correlation (a positive correlation can be considered as co-occurrence of the observed items). For example, BCT 2.4 Self-monitoring of behaviour tend to occur together with TB Tracking symptoms (rho = 0.84).

Are there differences in app characteristics between apps identified via Google Play search versus literature search?

In this work, mHealth self-management apps were found through two types of searches, a Google Play store search and a literature search. Willcoxon rank sum tests were performed on variables by search groups and Table 10 presents the statistics and correspondent p-values.

Table 10: Comparison between variables mean values between search types and correspondent p-values

Variables	W	pvalue
Stars	73.0	0.373
Ratings	164.0	0.015
BCT per app	137.0	0.142
TB per app	151.0	0.043
Actionability	89.0	0.720
Understandability	128.5	0.259

Interpretation

Significant differences were observed between Ratings (mean marketplace = 2940.77, mean literature = 119094.8; p = 0.015) and TB per app (median marketplace = 2, median literature = 3; p = 0.043) values.

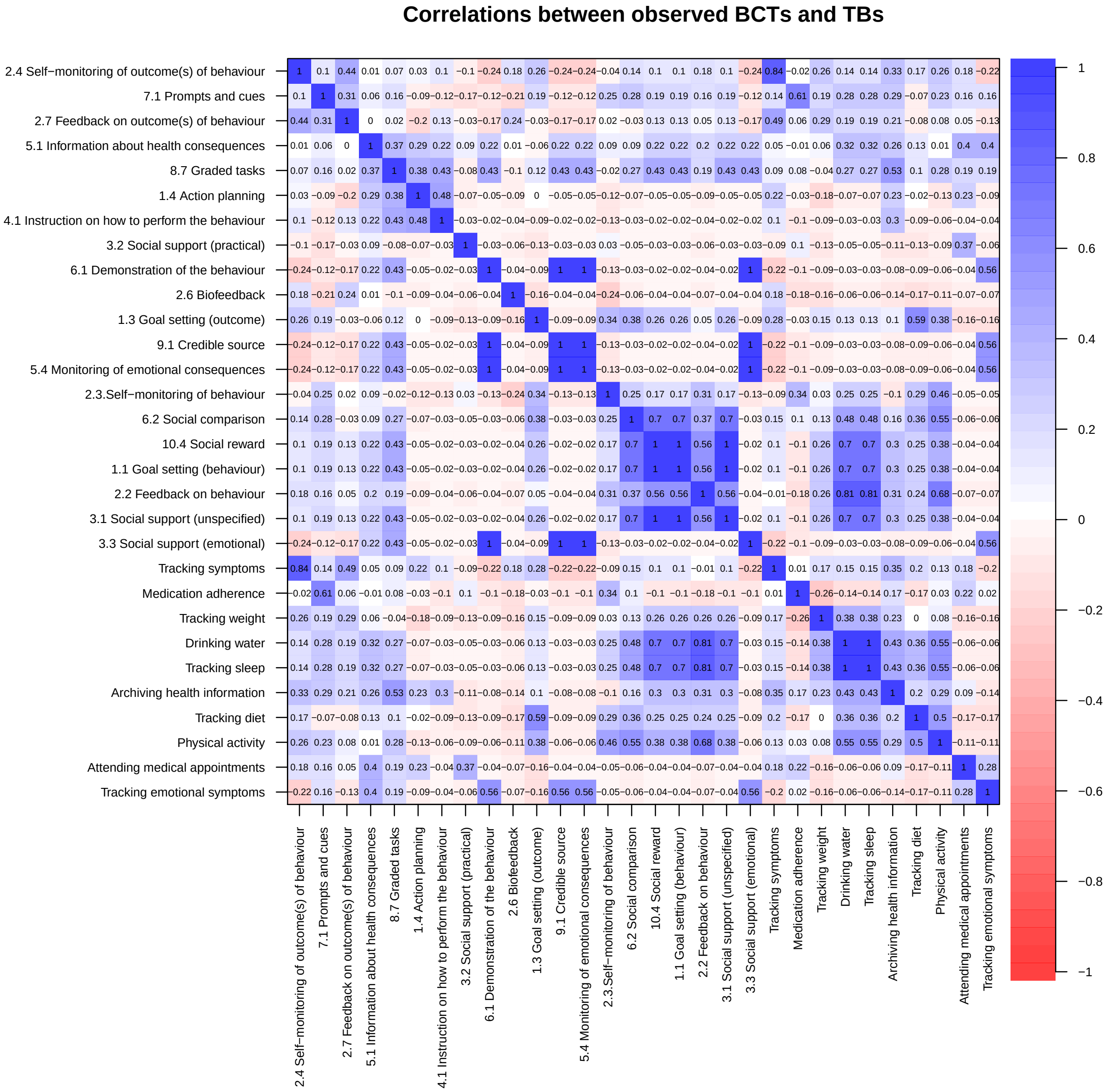

Figure 7: Figure 7: Heatmap of Spearman's correlations between observed BCT and TB
